# A Realist Review of How Community-Based Drug Checking Services Could Be Designed and Implemented to Promote Engagement of People Who Use Drugs

**DOI:** 10.3390/ijerph191911960

**Published:** 2022-09-22

**Authors:** Wendy Masterton, Danilo Falzon, Gillian Burton, Hannah Carver, Bruce Wallace, Elizabeth V. Aston, Harry Sumnall, Fiona Measham, Rosalind Gittins, Vicki Craik, Joe Schofield, Simon Little, Tessa Parkes

**Affiliations:** 1 Salvation Army Centre for Addictions Services and Research, University of Stirling, Stirling FK9 4LA, UK; 2 Canadian Institute for Substance Use Research, University of Victoria, Victoria, BC V8P 5C2, Canada; 3 School of Applied Sciences, Edinburgh Napier University, Edinburgh EH11 4BN, UK; 4 Public Health Institute, Liverpool John Moores University, Liverpool L2 2QP, UK; 5 Department of Sociology, Social Policy and Criminology, University of Liverpool, Liverpool L69 7ZR, UK; 6 The Loop, Unclassified Community Interest Company, Manchester M13 9PL, UK; 7 Humankind, Bowburn DH6 5PF, UK; 8 Public Health Scotland, Glasgow G2 6QE, UK; 9 Kinbank Social Research Consultancy, Tayport DD6 9AP, UK

**Keywords:** drug checking, harm reduction, substance use, drug intervention

## Abstract

With rising numbers of drug-related deaths in the UK and globally, exploration of interventions that seek to reduce drug-related harm is essential. Drug checking services (DCS) allow people to submit drug samples for chemical analysis and receive feedback about the sample, as well as harm reduction advice. The use of DCS is often linked to festival and/or nightlife settings and to so-called ‘recreational’ drug use, but research has also shown the potential of community-based DCS as an intervention serving more varied demographics of people who use drugs, including more marginalised individuals and those experiencing drug dependence. Whilst there is a growing evidence base on the effectiveness of drug checking as a harm reduction intervention, there is still limited evidence of the underlying mechanisms and processes within DCS which may aid implementation and subsequent engagement of people who use drugs. This presents a challenge to understanding why engagement differs across types of DCS, and how best to develop and deliver services across different contexts and for different populations. To explore the contexts and mechanisms which impact engagement in community-based DCS, a realist review was undertaken to synthesise the international evidence for the delivery and implementation of DCS. There were 133 sources included in the review. From these sources the underlying contexts, mechanisms, and outcomes relating to DCS implementation and engagement were developed and refined into seven programme theories. The findings of this review are theoretically novel and hold practical relevance for the design of DCS, with implications for optimisation, tailoring, and implementing services to reach individuals in different settings.

## 1. Introduction

Drug checking is a harm reduction intervention enabling people who use drugs (PWUD) to submit a sample of drugs for analysis. The primary aim of drug checking services (DCS) is to reduce the risk of harms to PWUD, including drug-related deaths, with potential for the associated reduction of harm to families, communities, and wider society [1]. To achieve this, DCS provide information about the composition of a tested sample to the individual who submitted it, coupled with harm reduction information and education [2]. The reach of DCS can extend beyond the individual submitting the sample, as services often provide warnings about particular drugs of concern in circulation, or share aggregated data on drug trends with a wide range of stakeholders for the purposes of harm prevention and reduction [3,4]. The most up to date global review of DCS cited 31 services operating worldwide in 2017, with the majority of these in Europe, and others recorded in North America, Central and South America, and Australasia [5]. Given the increase in drug checking literature since 2017, there has likely been an increase in services, but current exact figures are unclear. Services often have very different designs and modes of operation [5,6], and vary widely in relation to factors including: where they are located; how they are staffed; the equipment used and thus reliability and comprehensiveness of results; the time taken to provide results to individuals; how samples are submitted and results delivered; opening hours; the extent of ancillary harm reduction interventions also provided; and how they are funded [4,5,6,7,8]. This diversity of service provision creates challenges for the transferability and generalisability of knowledge about service effectiveness across contexts. 

One division of DCS type is between festival/nightlife DCS, which typically provide transient checking facilities at particular events [9], and community-based DCS, which are typically permanent, fixed-site services (with some services providing both forms of drug checking concurrently) [5]. Although there have been some examples of DCS in Europe catering to more marginalised groups of PWUD [5,10,11], the popular understanding and framing of DCS in Europe is as an intervention typically geared towards younger, ‘recreational’ PWUD [12]. Correspondingly, much of the literature has focused on drug checking as an intervention aimed at this demographic [8,13]. However, in recent years, and with the establishment of DCS in Canada [14] and elsewhere in North America [15,16,17,18] in response to the opioid overdose epidemic and other historically high levels of drug poisoning, there has been an increased focus in the literature on community-based DCS targeting other groups of PWUD. Despite this, the predominant framing of DCS as primarily an intervention for younger, ‘recreational’ PWUD in festivals, or within the nightlife settings, creates challenges for those seeking to evaluate the evidence base for community-based DCS which often seek to support people at higher risk of drug-related harm such as people who inject drugs [19,20].

Statistics on drug-related harm show why a better understanding of interventions, such as community-based DCS, which may provide effective support for PWUD at increased risk of harm, is important [19]. Drug-related deaths are at an all-time high, with 6189 deaths recorded across Scotland, England, and Wales in 2021 [21,22]. Across Europe, the most up to date figures show that an estimated 9200 drug-related deaths occurred in 2018, although this figure has been critiqued as likely lower than the true number [23]. However, as noted, differences between DCS in relation to context and setting limit the transferability of learning and evidence about best practice across DCS. Additionally, literature on drug checking has predominantly focused on evaluating the effectiveness of DCS as a harm reduction tool through its capacity to alter patterns of consumption amongst PWUD [3,24], with less attention paid to influencing factors on service user engagement. There are few case studies of DCS which outline how services in certain contexts operate, challenges faced by services, the change mechanisms that lead to the outcome of engagement, and the wider contextual factors which hinder or enable the implementation and operation of DCS [4,15]. 

To address this evidence gap, realist methodology was used to synthesise existing evidence for DCS implementation. This methodology is appropriate as causation is central to a realist approach: the premise being that the outcomes of a complex intervention programme, such as increased engagement, are directly caused by underlying generative mechanisms which have been activated in specific contexts [25,26]. Contexts can be wide ranging and comprise of individual, interpersonal, organisational, institutional, environmental, or geographical factors [26]. This causal relationship between contexts, mechanisms, and outcomes is referred to as the context-mechanism-outcome configuration (CMOc), and these CMOcs are described as the ‘programme theories’ of why a programme works. Each intervention will consist of a number of programme theories which can then be tested and refined. Through this process a better theoretical understanding of the intervention process is achieved, rather than simply deducing the effectiveness of an intervention, as typically seen within traditional systematic reviews. Further, the evidence surrounding drug checking is heterogenous, with several gaps in evidence and knowledge surrounding such facilities [6], and realist methodology is well suited to address this [27].

### Aims and Objectives

The aim of this realist review was to explore which mechanisms lead to increased service user engagement with community-based DCS, and how these mechanisms may vary by context. To achieve this, initial programme theories (IPTs) of what works and why, relative to community-based DCS, were developed, tested, and refined using both quantitative and qualitative evidence in the literature. The review questions were:

1. How has increased engagement of PWUD with DCS been evidenced in the existing literature?
2. What are the plausible mechanisms that increase engagement of PWUD?
3. What is the role of context in enabling or constraining engagement with DCS?

What context-mechanism-outcome configurations (CMOcs) best represent why diverse groups of PWUD may or may not engage with existing and future community-based DCS?

## 2. Methods

Unlike traditional systematic reviews, there are no standardised rules for realist reviews. However, the steps which Pawson and colleagues propose to guide the realist review process are: clarify scope of the review by providing aims and objectives; develop IPTs; search for evidence; appraise primary studies and extract data; synthesise evidence and draw conclusions; and disseminate findings [25]. These steps are iterative rather than sequential, and each stage may influence any other stage.

### 2.1. Formation of Initial Programme Theories (IPTs)

The first step of the review was the initial theory formulation about how community-based DCS have been developed and implemented internationally. This process provided the IPTs for the review about what works, for whom, and in what circumstances. The IPT development was led by WM and DF and involved discussion amongst the research team about what mechanisms may be linked to desired outcomes, and which contexts might be important for the intervention to work in. To aid with this process, existing theoretical frameworks such as the risk environment framework [28,29] and enabling environments [30,31] were drawn on. These frameworks seek to explain drug-related harm and harm reduction by exploring the interaction between individuals and environments and were deemed helpful for providing insight into the differing contexts and mechanisms that may influence engagement. Additionally, the Consolidated Framework for Implementation Research [32] was drawn on to provide insight into the processes through which DCS may be best implemented. This framework seeks to identify factors that influence complex intervention implementation and effectiveness so again was deemed useful for building IPTs about how to facilitate service user engagement. 

It is important to note that in order to provide the necessary breadth of programme theories which seek to explain why an intervention may or may not work, drawing on existing theoretical frameworks will likely not be sufficient [26]. Realist research typically requires researchers to hypothesise and build their own IPTs through layering existing theoretical frameworks as well as suggesting new CMOcs which are identified in the literature [26]. As well as developing IPTs through drawing on existing theory and literature, IPTs were developed from discussions during project meetings (made up of key stakeholders including: those with drug checking expertise; toxicology experts; public health and government actors; people with lived experience; and police), and from other relevant policy documents and reports. The IPTs were checked by other members of the research team (TP, HC, BW), and were refined and adapted until all were in agreement. Table 1 shows the themes that constituted the proposed IPTs. A detailed table showing the proposed contexts, mechanisms, and outcomes within the programme theories can be seen in Supplementary File S1. It is important to note that the IPTs acted as hypotheses and are often not completely representative of what is shown in the wider literature base. Therefore, the next step in realist methodology is to test and refine the IPTs as the data are synthesised and evidence emerges [25].

### 2.2. Search Process

To test and refine the IPTs, relevant electronic databases were searched in April/May 2021. These were OVID (NHS Scotland Journals; AMED; EMBASE; MEDLINE; PsycINFO); CINAHL; Health Source; SocINDEX; Scopus; Web of Science; Epistemonikos; Criminal Justice Abstracts; ScienceDirect; SpringerLink; Oxford Journal Online and SAGE Journals. Grey literature searches were conducted via BASE, Google, Twitter, and reports of known harm reduction agencies already providing DCS. Published thesis searches were made through EThOS. Hand searches of references sections of relevant articles were also conducted and authors in the field of harm reduction and drug checking were approached to ascertain whether any further literature existed that that had not been gleaned through formal searches. As DCS have evolved significantly since their beginnings over 30 years ago, the searches spanned from 2000 until present to ensure the most recent findings were included. Any systematic reviews or meta-analyses identified were also used for backward and forward citation tracking.

Initial scoping searches of the literature were conducted by two reviewers (GB, DF), using the following search terms:

1. (“Drug check *” OR “Drug test *” OR “Drug safety test *” OR “Pill test *” OR “Pill check *” OR “Multi-agency safety test *” OR “Street drug analysis” OR “Drug purity” OR “Drug market monitoring”)
2. AND (“harm reduction” OR “overdose” OR “substance use” OR “drug use” OR “toxicology” OR “spectro *” OR “chemometrics”)

Based on the results of the initial scope, systematic search terms used were as follows:

1. (“Drug check *” OR “Drug test *” OR “Drug safety test *” OR “Pill test *” OR “Pill check *” OR “Drug purity”)
2. AND (“harm reduction” OR “toxicology” OR “spectro *”)

Although the review has a focus on community-based DCS, the decision was made to keep the search string broad to best ensure that no potentially relevant evidence was missed during the search process. Additionally, whilst structured searches were conducted in April/May 2021, relevant materials which were highlighted to the authors after this period were included, up to and including February 2022 when the data extraction phase ended.

### 2.3. Inclusion/Exclusion Criteria

The literature included in the review consisted of the following study designs:

1. Experimental or intervention related studies including randomised controlled trials, prospective or other observational studies, case reports, feasibility/acceptability studies, implementation/evaluation/action or process research, quasi-experimental research, and qualitative methods;
2. Existing reviews of all types;
3. Grey literature of all types. The search for grey literature is justified by the goal of the realist review to seek out *‘the inner workings of interventions’* [25] (p. 29). Therefore, sources of all types that may contain important contextualisation of the intervention are deemed valuable.

Additionally, since this review was part of a wider project seeking to implement community-based DCS in three Scottish cities, notes taken from the wider project’s advisory group meetings were consulted to ensure the programme theories accurately described the underlying mechanisms and causal pathways of DCS which were discussed in meetings. Further, notes taken by the research team from two drug checking events/conferences, and notes from conversations with people already involved in drug checking, were also drawn on throughout the search and appraisal process to provide additional data representing current practice ‘on the ground’.

Whilst this review concentrates on community-based DCS, drug checking literature exploring other settings, such as festival or nightlife-based DCS, were included if data within the source was deemed relevant to the review questions and transferable to community-based DCS. Additionally, although the majority of included studies related to DCS, literature detailing other harm reduction interventions, such as injecting equipment provision or safe consumption sites, were not immediately discarded. In realist reviews sources can be included even if only a small section of text is relevant, hence the decision to include sources other than those relating directly to community-based DCS. Papers of this nature were included if they provided detailed information relating to ‘what works, for whom, and why’, and if they explored evidence gaps in terms of assessing contexts and mechanisms leading to increased engagement and implementation of a harm reduction intervention which appeared broadly comparable to DCS. Harm reduction literature which did not reference contexts, mechanisms, or outcomes that may have an impact on service user engagement and service implementation were excluded. Papers were not excluded based on intervention location or author location. Although findings of the review will seek to inform DCS implementation in Scotland at a later stage, inclusion of international studies was deemed beneficial given the small evidence base of UK-based DCS that would limit programme theory development. The review protocol was registered on PROSPERO (CRD42021246680).

### 2.4. Relevance and Rigour

Following the guidance set in the quality standards for realist reviews [33], each source was appraised for relevance and rigour. Relevance was assessed in relation to three criteria: population, intervention, and study design; explanation of context, mechanism, and outcome as individual aspects as well as in combinations; and explanation of theory. Studies are assessed for rigour in a different way from systematic reviews: standard quality assessment tools are not used as even papers that are deemed methodologically weak may provide insight to answer study research questions [34]. As advised in the quality standards [33], two researchers (GB, DF) conducted the inclusion/exclusion process and appraised and included a paper if it was deemed relevant and rigorous enough to be able to rely on the small section of data that was needed to inform the review. To ensure that the risk of bias was reduced during this process, a third reviewer (HC) checked a selection of included/excluded papers to ensure validity and consistency. Where there was inconsistency, a thorough discussion was held with additional members of the research team (WM, TP) to decide whether to include or exclude the data.

### 2.5. Data Extraction and Synthesis

Each eligible paper was read in full, and details on identified contexts, mechanisms, and outcomes within each included study were extracted and recorded in an Excel spreadsheet. Additional data including authors, jurisdiction, and type of intervention were also extracted. Data extraction and synthesis were undertaken by WM and DF simultaneously with one author per paper. However, to ensure consistency and validity of data extraction and to reduce bias, both authors cross-checked a quarter of the papers. All identified refinements for the programme theories were noted on the spreadsheet and discussed by WM and DF. Further, once the programme theories were refined from the data, members of the wider research team were consulted and provided feedback to further ensure that theories accurately described the underlying mechanisms and causal pathways of DCS.

## 3. Results

### 3.1. Search Results and Study Characteristics

**Figure 1:**
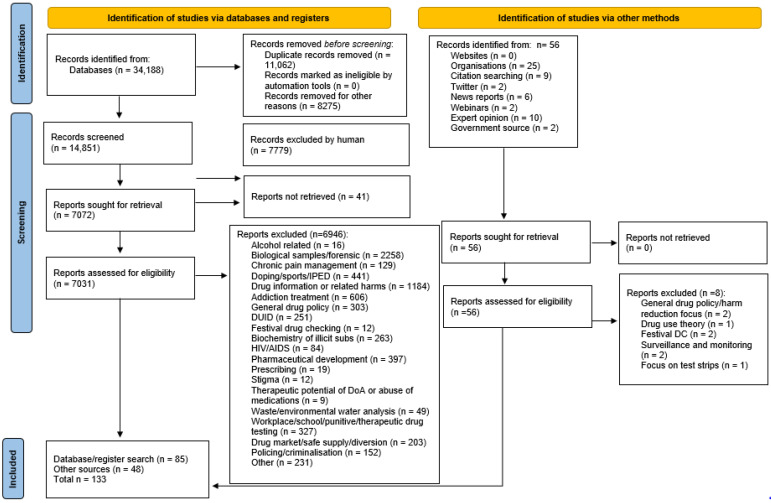
PRISMA flow diagram.

In the first stage of searching, after removing duplicates, 14,851 titles and abstracts were screened against the inclusion and exclusion criteria. 7072 sources were retrieved an assessed for eligibility, with 6946 excluded as shown in the PRISMA flow diagram (Figure 1). The assessment process was carried out concurrently by two members of the researcher team (GB, DF) with any differences in exclusion/inclusion discussed. Sources were deemed eligible for inclusion if they contained data either directly related to, or relevant to, community-based drug checking. Excluded sources were categorised as shown in Figure 1 below. Eighty-five sources obtained through the search process were assessed as eligible for inclusion in the review. A further 56 sources were identified outside of the search process, via additional methods (Figure 1). Of these, 48 were assessed as relevant to community-based drug checking and suitable for inclusion in the review. Therefore, a total of 133 sources were included in the review. Key characteristics of all included papers were recorded, including which programme theories each source supported (see Supplementary File S2).

### 3.2. Testing and Refinement of Programme Theories

The development of IPTs into seven refined programme theories is shown in Table 2 and detailed below. The programme theories are represented by headings which the wider research team agreed best describe their core concept. As previously discussed, the premise of realist research is to highlight the causal relationship between contexts, mechanisms, and outcomes. Therefore, all programme theories are written with this in mind and explicitly describe how various contexts lead to specific mechanisms which, in turn, lead to the desired outcome of increased engagement with DCS.

#### 3.2.1. Legislation and Regulation

*Sub-theory 1: Exemptions and service user risk*

For the implementation and running of DCS, the legal framework of the country was described in the literature as a clear contextual factor, particularly in relation to risks experienced by the service user. For example, in some US states, paraphernalia laws create barriers to implementation and decrease the likelihood of service user engagement [15,18,35,36]. In other jurisdictions, such as the UK and Canada, DCS may be able to operate within the law or receive ‘legal exemptions’ which will enable services to operate without fear of disruption from police [37,38]. Despite this, individuals travelling to or from the service were described in the literature as still being subject to potential criminalisation [39]. However, the extent to which those using DCS are at risk of being criminalised in practice varies widely across contexts and is influenced by a range of factors such as policing practices and service user demographics [40]. Conversely, there are examples of legal frameworks which provide more robust protection for DCS service users such as in New Zealand where drug checking is now explicitly legalised [41]; Portugal, where personal possession is decriminalised [42]; and Italy, which passed legislation allowing drug checking at local state level [38]. In the Netherlands, there is an agreement with the public prosecutor that people will not be charged when entering or leaving DCS [42,43].

Importantly, if DCS operate within a protective context, there appears to be less fear of criminalisation reported by service users [7,19,43]. Reduced fear was described an essential mechanism for service user engagement, with 60% of respondents in one study stating that their main concern around using DCS would be criminalisation [44]. Service user fear around legal repercussions was echoed in multiple studies [19,20,35,39,45,46]. If there are high levels of fear around criminalisation, this seems to outweigh the perceived benefits of drug checking [47,48,49], and PWUD have been described as *“not always prepared to play the odds”* [50] (p. 1553):

*“What’s to stop them from stopping us when we leave here? Right? To see if we finished all our dope and charging us with possession, right? You know, just leaving here, is that a probable cause for them to stop us?”.*

[39] (p. 5)

According to the literature, only through enabling legislation or clear exemptions will there be a reduction in fear and a subsequent increased likelihood of engagement with DCS. Without this, fear of criminalisation remains a significant barrier to engagement [39,40,51]. Indeed, in Barratt et al.’s study (2018), over 94% of participants said they would not use a service if arrest was a possibility [7]. Despite the introduction of exemptions and protections for service users, increased engagement may not be immediate as there may be a time lag between such developments and reduction of fear [40]. This may be particularly true for marginalised groups that have a history of negative interactions with police [39,52,53].

*Sub-theory 2: Exemptions and staff risk*

As with the programme theory above relating to service user risk, literature depicted that there must also be an enabling legislative framework, or legal exemptions, to provide assurance that DCS will be able to operate without disruption and staff will not be criminalised. Measham (2019) describes the legislative context in the UK which asserts that encouraging or assisting a crime constitutes a crime in itself [2]. Therefore, given the illegal nature of drug possession, the legal risks experienced by DCS staff may be substantial. In the UK, staff exemptions through Home Office licences may cover the handling of controlled drugs, but not the advice given by DCS staff. This means that staff could still be subject to risk of encouraging or assisting future offences [37]. However, as discussed in the programme theory above, some DCS in the UK have navigated this challenge and operated without Home Office licences through explicit agreements with supportive local police forces, national police chiefs, and the Minister of State for Policing and the Fire Service [38,54]. Outside the UK, exemptions to protect staff were discussed relative to other countries such as Australia where the Courts and Legal Services of New South Wales state:

*“For a person to be aiding and abetting the offence, they must be ‘linked in purpose’ with the drug user, and that it is also necessary for the person to engage in some action or encouragement which makes the offence more likely to occur”*.

[55] (p. 15)

Thus, because DCS does not fit into this description, staff can act without fear of arrest in Australia [55]. In other jurisdictions, however, the lack of clear legal standing of services can be a significant contextual barrier [16,35,36,48,56]. Carroll et al. (2022) noted that, without enabling legislation or exemptions for staff, *“harm reduction organisations are equally vulnerable to the misguided, punitive responses so often directed at the people they serve”* [15] (p. 10). In some countries, legislation prohibits service staff from coming into direct contact with drugs. In others, even the presence of drug checking equipment places staff at risk of being criminalised for allegedly facilitating drug consumption [14,16,35,36,57,58]. Additionally, another necessary contextual factor identified through the literature was the need for a supportive police culture relating to harm reduction, rather than an enforcement-led approach [2,42,59,60]. Even in contexts with enabling legislation or exemptions, this cannot be assumed a priori [40,61]. There was some evidence that police may still take a penal approach in such contexts which is at direct odds with the aim of harm reduction [62]. This was particularly evident when exemptions and legislation surrounding DCS were unclear [15].

Where clear legislation or exemptions exist, as well as demonstration of public health-focused policing practice (discussed in sub-theory 4), evidence indicated that staff have more clarity around how DCS can feasibly operate [16,18,60]. In turn, fear of criminalisation is reportedly eased [43,55]. Increased partnership working and communication between services and police were said to be key factors which increase staff clarity and reduce fear of prosecution [60]. Indeed, staff in such contexts were reported to be more likely to engage with the police to come to effective local arrangements and practices surrounding services which appears to result in more effective implementation of DCS in the longer term [43,60].

*Sub-theory 3: Government and policing policy*

New Zealand was the first country in the world to explicitly legalise drug checking through the Drug and Substance Checking Legislation Bill [41]. As previously discussed, other locations have provided legal exemptions for DCS that allow them to operate [13,37,63,64,65,66] However, unclear service legislation or exemptions hinder implementation of harm reduction services [36,58,67]. The literature also asserts the need for local police divisions and/or prosecution services to retain the ability to make formal or informal agreements relating to DCS [42,59,60]. A policy environment where local police divisions have considerable autonomy in their response to drug-related issues was framed as a key contextual factor enabling the development of such agreements. Devolved policing structures, such as the strong local operational independence of the English policing structure, can create opportunities for bottom-up reform [59]. Indeed, support from local policing divisions has been central to enabling the implementation of DCS in the UK in both festival and community-based settings [2,37,38,54]. Without such arrangements/agreements, challenges relating to policing practice, such as increased stop and search in areas surrounding services, can act as barriers to engagement [15,16,42,52,61,68].

A policy environment consisting of transparent support for DCS, specifically from those in government, from high-level policy and public health actors, and from high-level police ‘champions’, was described as necessary to facilitate the mechanisms of wider public support and increase perceived legitimacy of services [16,38,43,58,59,60,69,70,71]. Although a growing number of police and crime commissioners across England are reportedly championing a harm reduction approach to drugs, this support remains inconsistent at a UK government level [59]. There also continues to be a level of *“government reticence”* towards DCS in other countries such as Australia [72] (p. 1), and variations in support between regional governments [7]. Conversely, across Canada, DCS have both the support of the federal and provincial governments, as well as explicit support from high-ranking police officials in some jurisdictions [1,58,64]. Within an enabling policy and political environment, this reportedly allows for more autonomy for local police divisions [38,58,59] and better understanding and acceptance of how DCS can work within existing police roles and responsibilities [42,60,68]. Within such enabling contexts, local police officers have been described as feeling more protected in their decisions to interpret the law through the lens of public health [52,59,68]. Indeed, drug policy introduced for UK festivals states that police must protect the public from harm, and that, in this environment, drug checking plays a role in meeting this aim [73]. As a result of increased autonomy, clarity, and understanding of how DCS can be implemented in a way that still supports police roles and responsibilities, there exists increased likelihood of successful local agreements [37,38,59], better partnership working and relationships between police and DCS, and between police and the general public [60].

*Sub-theory 4: On the ground policing practice*

The majority of the data informing this programme theory were drawn from policing practice relating to harm reduction services more generally. However, given the impact of existing policing practice on these services, it is likely that DCS will operate within similar contexts, and with similar mechanisms leading to engagement. A key contextual factor discussed in the literature was the need for existing positive relationships between local police and harm reduction services. Without this there have been circumstances where police officers have voiced intent to disrupt harm reduction services if they were perceived as interfering with police agendas such as maintaining public order [15,16,40]. Along with positive relationships between police and services, data suggest that there should be limited use of enforcement-led policing in the areas surrounding DCS [60]. Indeed, numerous international studies demonstrate that policing practice around harm reduction programmes, such as increased patrol, stop and search, confiscation of drug paraphernalia, dispersal orders, and possession charges, can undermine the engagement of PWUD with such services [15,39,40,42,52,74].

Policing practice can be particularly problematic for populations where fears are related more widely to history of negative policing practices towards certain marginalised groups [39,52]. For example, there are stark disparities in policing practices experienced by people who use drugs from Black, Asian, and other global majority ethnic backgrounds [48,75]. Indeed, police responses to individuals using DCS may vary by service user demographic and the area in which DCS are located [39,40,42]. Services accessed by marginalised individuals may cause greater concern around potential disorder in the vicinity of services relative to those generally accessed by people who are more socially integrated [38,43,76]. Studies discussed the need for harm reduction training to be embedded in police forces at an institutional level, prior to implementation of interventions like DCS [59,60,68]. Attending events and training organised by drug charities and harm reduction advocates could allow police officers insight into *“causes, consequences and complexities of drug use, and increase understanding of users’ health and social needs, and engender scepticism or nuance in attitudes towards criminal justice approaches”* [59] (p. 529).

With changes in policing practice away from enforcement-led approaches, there appears to be a reduction of fear and stigma, key mechanisms in this programme theory [20,40,43]. Further, positive police relationships can also increase trust held by services themselves [38,60]. These mechanisms are reportedly essential because otherwise the perceived risk of engaging in DCS can be deemed to be higher than the perceived risks of taking the drugs without testing [19,47,50,51,77,78,79]. Other harm reduction interventions, such as safe consumption sites, have reported that presence of police around services, and practices such as stop and search, also present barriers to engagement [39,42]. Moreover, such policing practices may encourage individuals to use drugs on their own, or in secluded areas, rather than access services, all of which are linked to increased drug-related harm and deaths [77].

#### 3.2.2. Existing Drug Market and Level of Drug-Related Harm

The level of drug-related harm, or concerns over emerging risk related to the unregulated market, was described in the literature as a key context in the implementation of DCS. Globally, PWUD are facing increasing risks concerning adulteration, mis-selling, and high potency drugs in circulation [11,23,80,81]. The starkest example of such dynamics is the ongoing opioid overdose epidemic in North America [16,17,47,63,82,83,84]. A major driving factor of this epidemic is the increased presence of fentanyl and its analogues in the drug market. Although the increase in drug-related deaths has been particularly steep in North America, they are also rising in the UK, Australia, and several countries in Eastern and Western Europe [2,37,85]. Scotland has the highest rate of drug-related deaths and harms in Europe [22,67], driven in part by the increasing prevalence of ‘street’ (novel) benzodiazepines which can vary considerably in content and strength [38]. Within the context of increased drug-related harm, vocal concern from communities, policy, and research actors, and governmental support for harm reduction, were also identified as contextual factors which can present opportunities for the implementation of DCS. The review literature evidenced growing activism, research, and action by a broad range of stakeholders, for example by existing harm reduction services, as necessary to secure funding and provide some level of DCS for service users [37,49,65,71,82,86,87,88]. Government support (or otherwise) can impact the likelihood of new interventions being implemented and can affect important aspects of operation such as the scale of DCS in a jurisdiction or funding received [6].

Within the contexts of increasing drug-related harm, there is growing acknowledgement amongst a range of stakeholders that PWUD are increasingly exposed to health risks [11,13,85]. This appears to provide opportunity to work towards implementing novel harm reduction interventions, creating pressure to act. For example, in Canada there has been a *“growing investment in legislative and policy change”* in order to *“scale-up […] evidence-based treatments and interventions”* [78] (p. 2), including safe consumption sites, DCS, and take-home naloxone [47,89,90]. Although varied across states, the US has seen a similar pattern of growing acceptance of the need to implement novel harm reduction interventions [58,65,91]. In a Scottish context, discussions around drug checking have been part of a wider conversation around the need for implementation of a systemic harm reduction approach, including the implementation of safe consumption sites [38,42,67]. A further mechanism in the context of increased drug harm and conversations around DCS is a growing understanding of the evidence base regarding drug checking as a market monitoring tool [2,3,13,38,64,72,92,93,94,95]. There are examples of DCS detecting shifts in market trends and providing early warnings when substances of concern are in circulation [96,97,98].

As a result of increased understanding and motivation, studies show that support for DCS is growing amongst a wide group of stakeholders including governments, policy actors, and public health bodies [11,24,58,84]. This is also evidenced by a substantial growth in the number of DCS globally in recent years, with services across Europe, North America, Australasia, and South America [6]. In Canada, three services (across Vancouver, Victoria and Toronto) are now federally funded as part of a five-year pilot in response to the drug death crisis [99,100]. In the US, while implementation has been slower, there is evidence of growing support from state governments and public health actors [15,18,65,87]. For example, the Centre for Disease Control has voiced support for DCS, and a number of states are introducing legislation to allow some form of drug checking provision [15,16]. In the UK, although there has been a lack of either explicit support or objection from the UK Government, there is evidence of growing support amongst a range of stakeholders including public health institutions and some local police divisions [2]. There have been pilots in England supported by a number of stakeholders [2,37,54].

#### 3.2.3. Integration into an Existing Service

*Sub-theory 1: Service staff point of view*

Integration of DCS into an existing service with a core harm reduction ethos was described in the literature as a central context for implementation, since abstinence focused recovery services were cited as less compatible with the objectives of drug checking [11,15,38,58,63,76,88]. A harm reduction ethos is described as staff having non-judgmental attitudes and a deep understanding of harm reduction practice and its underpinning logic [19]. However, existing services must have capacity (space, time, and resources) to accommodate the integration of DCS, as well as capacity to provide DCS-specific training for staff [76,89,101]. Stakeholders in harm reduction services have noted concerns around the staffing and resources required to perform drug checking, particularly if utilising more advanced techniques [101]. As noted above, services also require a good pre-existing relationship with the police to facilitate service implementation. There is also evidence that integration into existing drug services can ease police concerns and lead to DCS being seen as more acceptable [38].

If DCS operate in the service contexts described, the literature indicates that staff have an increased willingness to learn about drug checking and the ways in which it can be integrated into existing service processes to better meet service users’ needs [15,53]. Indeed, staff can perceive DCS as a tool for engagement and relationship building within existing services [86,90,101]. As a result of increased staff willingness to learn about DCS and its implementation, there will reportedly be better leveraging of staff knowledge, with staff keen to share expertise and enhance practice [53,64]. Staff in existing services will often have extensive knowledge of the service landscape, providing a potential infrastructure for drug market information to be shared across the service landscape and amongst PWUD [43,102]. However, there is currently a lack of data showing how staff in DCS share trend information with service users, other services, and wider stakeholders. There is therefore a need for further exploration of how the information is presented, whether it reaches intended target groups, and how it is received and understood.

Integration of DCS within existing services can reportedly facilitate improved relationships between service users and staff across services [15,53], and aid in the provision of a more holistic approach and wrap-around care. This reportedly creates greater capacity for service users to be linked into additional supports since *“staff know the area and what is available locally”* [37] (p. 13). This is particularly key for individuals who have become ‘disconnected’ from care and services [15], or who have never been in contact with services, where drug checking may act as a ‘hook’ for engagement [103]. Sherman et al. (2018) noted that:

*“Harm reduction counseling, health education and connection to services including treatment for substance use disorder should be part of any drug checking program”*

[83] (p. 11)

*Sub-theory 2: Service user point of view*

Integrating DCS into services that people already access for other drug interventions and supports is noted as important for engagement [11,15,82,83,104]. However, this will typically differ by target group. For example, those using drugs in a ‘recreational’ capacity may not require DCS to be integrated with other trusted services, as a significant proportion of this group will solely use DCS and will never have been in contact with any other services in relation to their drug use [2,12,43,54,102,103,105]. However, services aimed at more marginalised groups tend to be integrated into low threshold settings offering other harm reduction interventions such as injecting equipment provision, take-home naloxone, relational support, and support with housing and health [15,63,64,76,83,88,100,106,107]. Even if individuals are accessing existing support within a service, literature indicates that they must see the value in and purpose of DCS in order to engage. Potential service users may not see the purpose of DCS due to: a lack of interest about knowing the composition of a sample [108]; expectation of contamination, for example if a drug such as fentanyl is highly prevalent with the market [108]; ambivalence to overdose [20,50]; having trust in their supplier [109]; being in withdrawal; and the time investment and requirement to give up drugs for testing [20]. Additionally, whilst integration into a particular service may increase engagement amongst some PWUD, it may act as a barrier to engagement for others [19,38]. For example, DCS integrated into supervised injection site may act as a barrier to engagement amongst wider groups of PWUD, who may be reluctant to access settings specifically for people who inject drugs.

If service users see the value of drug checking within the settings they are already utilising, then they will reportedly be more interested in engaging with DCS whilst accessing other interventions. This increased willingness to consider the service is the key mechanism within this programme theory. For example, a study on the integration of drug checking in a harm reduction site in Bordeaux, France found that over half of the service users were willing to utilise drug checking whilst accessing a range of other supports [11]. It is worth mentioning that, in a Scottish context, although there are currently no safe consumption sites nor DCS, one study found that 75% of surveyed people who inject drugs would be willing to use a safe consumption site [67]. Although willingness does not necessarily translate into actual use, it does show potential for increased footfall for people who inject drugs accessing existing integrated services, and this may lead to increased willingness to consider using DCS as well. Further, if individuals are already accessing the service, literature suggests that they may feel less stigmatised, more accepted, and value their pre-existing relationships with staff [56,76,79,88,110].

Due to the complexity of the risk environment for PWUD, and a range of factors often external to the site itself, there is a need for further evaluation of outcome data on how engagement across different sites varies according to the contexts and mechanisms outlined. However, available evidence in the literature strongly suggests that increased trust due to existing non-judgmental relationships with staff, and increased willingness to consider using a DCS due to accessing multiple interventions in one site, are beneficial mechanisms to engagement [11,15,58,64,82,83]. It appears that this is particularly applicable for those with multiple, intersecting vulnerabilities [19,38,64,110].

#### 3.2.4. Lived/Living Experience at All Levels

Having people with lived or living experience (‘peers’) central to the running of a DCS has been identified as an essential context to providing a health-equity approach [111]. This is not a novel concept, and the importance of lived/living experience in the running of services has been shown in other harm reduction initiatives [56,89]. Despite this, DCS have at times been described as being gatekept in terms of expertise needed [58], and those with lived/living experience are often in support roles. For increased service user engagement, the concept that people with lived/living experience should be limited to being peer researchers, or working in support roles, should be challenged where possible [58]. If a service is to be community-based, it must provide a meaningful, inclusive approach with families, friends, carers, and PWUD at the centre of design, analysis, interpretation, and knowledge translation activities [16,19,89,111]. This may, at times, be in tension with the priority of some stakeholders (e.g., government) in managing risk through regulation, for example by preferring trained health professionals rather than peers [38,43]. Concerns around insurance and challenges relating to increased testing times, and/or different interpretations of results across peers and chemists, may pose barriers to lived/living experience involvement. However, international evidence highlights instances of peers being trained in the operation of DCS equipment and interpretation of results [16,58]. In particular, there is evidence that highly marginalised communities, such as people who inject drugs, may be more likely to engage with DCS if they see themselves represented in the DCS staff [19,53]. However, evidence is also clear that people with lived/living experience must receive ongoing support and protection due to potentially high levels of stress associated with working at DCS. Particularly for peers working in contexts with high levels of drug-related deaths, issues such as trauma, post-traumatic stress disorder, and grief have been reported [89], and *“such work could have a negative impact on staff in recovery”* [101] (p. 529).

If the DCS operates in the contexts mentioned, PWUD may perceive the service to be more inclusive and responsive [1]. This mechanism has also been described as a sense of ownership around a service [89]. Further, services will reportedly be viewed as more trustworthy, reliable, and credible by PWUD and their families/friends [35,64]. Indeed, Wallace et al. (2020) describe prospective service users as saying that, in their opinion, the presence of lived/living experience staff makes the service feel like a safe place to attend [19]. In particular, PWUD report feeling more comfortable discussing personal information with lived/living experience staff, as well as sharing insight into issues such as current drug market trends, and what facilitators and/or barriers there might be to future engagement, increasing the knowledge available to DCS [56]. As well as uncovering issues around design and delivery which may not otherwise be addressed, increased knowledge exchange by people with experience of drug use may also enable discussion about how to recognise and understand different ‘feelings’ of drugs, potentially enhancing and better contextualising harm reduction education [16]. The expertise of PWUD relating to drug use is often discredited as untrustworthy or otherwise invalid [16]. However, people who have experience of drug use may be best placed to identify potential trends and provide insights, in comparison to people with no first-hand experience [112].

#### 3.2.5. Accessibility

Many papers discussed the importance of an accessible service, but the meaning of accessibility differed across articles. In some studies, service location was described as central to accessibility, noting that services should be central to where PWUD live and congregate [3,20,39,76,77,108]. As noted by McCrae et al. (2020):

*“Physical distances between harm reduction services and the residences and drug purchase and use locations of PWUD have an impact on their willingness to access such services”.*

[77] (p. 4)

As it is challenging for single, fixed-site DCS locations to be central and accessible for all PWUD in a jurisdiction, the availability of drop-off points in various locations and postal drug checking was also discussed as a potential means of increasing accessibility [6,19,55,77,79,107]. Some research discussed the use of outreach in ensuring accessibility of a service, for example by use of mobile testing units [18]. However, an important contextual factor relating to this theory is whether the legislative landscape of a country allows such outreach testing or sample collection. In the UK, collection and transportation of controlled substances is illegal under the Misuse of Drugs Act 1971, unless by police approved couriers [38]. As a result, outreach in relation to mobile testing and/or collecting samples may not be possible, particularly within community settings. However, outreach work could still be utilised by DCS as a means of spreading awareness of fixed-site services, transporting people to and from services, distributing drug trend information, or providing test results [38].

If services address contextual factors relating to accessibility, they are typically considered to be more flexible [101] and more inclusive [19]. Additionally, when individuals have the option of choosing to engage with outreach, or using a discrete drop-off point, rather than attending a fixed site, this has been said to support those who wish to use DCS with more anonymity [38,58,76]:

*“There’s business people out there, there’s lawyers out there that use drugs. They’re not going to go to a place that let’s say people who are homeless would go to”*.

[19] (p. 4)

Betzler et al. (2020) echo this, discussing how discreet spaces for dropping off drugs are viewed particularly favourably by people who have concerns about anonymity and data privacy, and who believe they have a lot at stake by engaging in illegal activities [44]. However, it is important to note that drop-off points and/or outreach can reduce the capacity of services to offer on-the-spot testing, particularly if legislation prevents mobile testing, so this may be less attractive to individuals who favour quicker results [38].

The belief that services are flexible, accessible, and provide a choice of how to engage, has been argued as potentially resulting in a higher number of service users being reached overall, although there is a lack of robust outcome data on this issue [20,38,76,77,78,108,110]. Outreach sample collection, or testing in particular, may increase engagement amongst those with wider support needs relating to high-risk drug use such as injecting [37,58,77], those who are rough sleeping, and those with more entrenched patterns of use [19,38,54]. For example, McCrae et al. (2020) suggest in-reach into supported accommodation/hostels as a potential means of increasing engagement amongst such groups [77]. Equally, outreach models have been suggested to provide better access for rural communities [38,79]. However, it is important to note that the majority of these reported outcomes are anecdotal, largely due to challenges with securing outreach funding and concerns around legality. There is a need, as outreach becomes more established within DCS, for more in-depth exploration of quantitative engagement outcomes, backed up with qualitative work, exploring reasons for/barriers to engagement. Moreover, it will be important for research to explore how different means of expanding accessibility (e.g., postal, in-reach to hostels, mobile checking, drop-off points) may impact on engagement amongst different groups of PWUD.

#### 3.2.6. Testing Process

*Sub-theory 1: Equipment and expertise availability*

The context of drug market dynamics reportedly dictates the equipment and expertise required to operate DCS in a particular jurisdiction [5,14,17,18,69,95,107,113,114,115,116,117]. For example, the development of testing methods in North America has largely targeted optimisation of the detection and quantification of fentanyl and its analogues [114,118,119]. In the UK, The Loop pioneered the use of Fourier-transform infrared spectroscopy (FTIR) for identification of 3,4-Methylenedioxymethamphetamine (MDMA), ketamine, and cocaine at event-based drug checking from 2016 onwards [2]. In the Netherlands, DIMS has focused on developing methods using FTIR to quantify MDMA, ketamine, and amphetamine, which are reportedly among the primary expected drugs submitted for testing there [43]. In a Scottish context, the high prevalence of novel benzodiazepines [120], which can vary significantly in potency, is necessitating consideration of equipment and methods suitable for their identification at point-of-care [38].

The literature reports that all services must consider trade-offs between comprehensiveness and reliability of results; result turnaround time; and cost of equipment and staffing [7,95]. More technologically advanced equipment may be prohibitively expensive for services unless they receive substantial funding [5,17,38,43,62,94,95,101,113]. Indeed, the availability of funds and expertise to enable method development, including capacity to perform laboratory-based confirmatory testing to validate point-of-care methods [97], has been noted as an essential context for services being able to provide more detailed and comprehensive results, and more responsive to emerging drug market dynamics [18,64,99,115,119]. However, many services operate without significant government funding [6,15,56] meaning that they may have to: *“rely on less precise testing methodologies and equipment due to lack of funding or support”* [95] (p. 2). Partnerships with universities and other DCS may help provide more affordable opportunities for technical development for DCS operating without significant government funding [5,15,58].

Level of staff expertise was described as an important contextual factor in the operation of DCS [19,38,58,84,113,117,119,121]. The level of expertise and training required varies by equipment type, sample complexity, and the comprehensiveness of results that services aim to provide [95]. Several sources indicated the need for DCS to employ staff with expertise in drug checking equipment and/or chemistry in order to interpret complex results with sufficient accuracy [2,11,53,56]. This initially appears contradictory with the programme theory relating to lived/living experience involvement and therefore highlights the necessity of a multidisciplinary staff team and intensive training for all staff. However, due to limited resources, a number of studies described how services often have to rely on training existing staff [11,15,101,121]. Links to drug checking networks are noted as helping staff deal with equipment challenges by enabling them to draw on the significant expertise of others in the field [15,58].

Within enabling contexts, such as existing staff expertise and available equipment, staff will reportedly have greater capacity to interpret results, will feel that they have adequate resources to conduct the service, and be more confident in its implementation and operation. However, this varies significantly across jurisdictions and services. One study of an emergent DCS operated by staff without prior drug checking expertise noted significant technical challenges and staff frustrations around equipment and its limitations [15]. Another study exploring the perceptions of harm reduction stakeholders found that the margin of error relating to the concentration of active drugs would need to be low for them to feel comfortable delivering DCS [101]. These two studies aside, there is limited focus in the literature on staff perceptions or experiences, and there is a subsequent need for greater understanding of such issues, particularly in cases when implementation occurs in services where staff lack initial expertise.

In terms of outcomes, with appropriate equipment and methods, and trained, competent staff, the literature suggests that DCS should be able to provide sufficiently accurate and detailed information to service users. However, DCS often face challenges in relation to: equipment providing questionable results [15,16]; identification of all substances in a sample [95,117]; identifying substances in low concentrations [96]; testing of complex, multi-component samples [11]; the ability to consistently provide quantitative information [69]; and the rate of false positives and negatives [114,122,123]. Services which provide more comprehensive results and mitigate some of these challenges tend to: receive significant funding; have drug checking experts integrated in the staff team; and have capacity for continuous method development [2,5,13,43,58,97,107,115,119].

*Sub theory 2: Service user expectations of DCS tests*

As noted, point-of-care drug checking technologies have several limitations which vary by factors such as those listed in the above programme theory [6,11,16,56,62,66,95,96,115]. It is therefore vital for services to communicate these limitations to service users [107,110,124]. The importance of particular testing limitations may vary according to context or service user’s substance use. For example, in places where fentanyl dominates the market, methods which only screen for the presence of fentanyl (such as fentanyl test strips) may be of limited utility to those using other opioids [63,114]. However, in a different location where fentanyl is not common, or in the same location for non-opioid samples, fentanyl screening methods may hold more value [58,79,125]. Furthermore, not all DCS are able to give immediate quantitative information at point-of-care [64]. Services which do not have quantitative capacity may send samples for more comprehensive laboratory-based testing, with longer turnaround times [6]. However, studies have found that the waiting time to receive results is a key barrier to engagement, particularly among people who use drugs daily [20,48,90]. Additionally, communication of results should inform a broader harm reduction conversation focusing on issues such as: dosage and safer consumption; poly-substance use; and interactions between prescribed medications and illicit substances [37,53,95,126]. This can help offset the limitations of testing by situating the test result in a broader process of harm reduction support and education and may address concerns of stakeholders that DCS may provide a false sense of security [2,43].

If a DCS operates in the contexts of clear communication and transparency and provides results which, to some extent, are able to meet service users’ priorities, then this is said to lead to higher levels of satisfaction with the service since the test results, and their limitations, will be better understood [38,56,86]. Service users having a better understanding of drugs and related risks, and of achievable means of mitigating these risks, can lead to a greater sense of control [1,16,86]. The information provided through DCS can lead to increased risk/drug literacy and can inform behavioural outcomes including: discarding of a sample of concern [3,54,105]; returning a drug to a supplier or informing them of the results [2]; sharing information with other PWUD [110]; using a smaller amount, using over a longer period, or mixing less [24,91]; taking drugs in the presence of others and carrying naloxone [46]; or changing mode of administration [1]. There is evidence that the provision of quantitative results (e.g., drug concentration) can increase engagement [14,108,114]. However, there is also evidence that PWUD are still willing to engage in DCS even where results are not comprehensive and there are limitations, as long as these limitations are clear and the service user still believes the test to be useful [5,7,10,35,45,46,49,65,87,91,104].

#### 3.2.7. Service Users’ Previous Experience

An important individual level context mediating the use of DCS was said to be whether a person has had previous negative experiences associated with drug use, such as overdose or other adverse effects, or if they have witnessed the negative experiences of others [17,78,82,103,104]. Some individuals may “*perceive themselves as susceptible*” to overdose based on past experience [48] (p. 6). Beaulieu et al. (2020) found that witnessing an overdose was positively and significantly associated with the use of DCS [78]. Additionally, those who had training in harm reduction, or experience and knowledge of DCS, were reportedly more likely to use them [24,82]. Importantly, after training, people who had no previous experience of overdose were also increasingly likely to use drug checking methods such as fentanyl test strips [104]. One of the mechanisms behind this was said to be an increased fear due to greater appreciation of the risks stemming from an unpredictable drug supply [1,45,49,57,78,104].

As well as increased fear, another related mechanism relative to this programme theory was said to be increased awareness of the potential risks associated with ongoing and/or future drug use [19,35,37,48,81,103,104,110]. Mistler et al. (2021) found that those who have experienced non-fatal overdose have a particularly heightened risk perception [110]. A third mechanism was described to be an increased desire to know the content of a drug [12,35,82,127]. In response to such mechanisms, individuals are possibly more likely to engage with DCS. One study described this as people taking *“prevention into their own hands”* [110] (p. 5). Further, studies report that service users can encourage engagement amongst other PWUD [2,57,104].

Disconfirming evidence existed in some of the literature, however. Some studies found that engagement may be lower for those with longer histories of drug use as they may perceive themselves to have higher drug literacy and be less susceptible to harm, or be used to managing risk themselves [20,54,92,102,123]. Given this is a direct contradiction to the findings reported thus far, an important caveat to this programme theory seems to be that additional contextual factors will mediate outcomes on a case-by-case basis. Individual level contexts, such as being in withdrawal and ambivalence to overdose, have also been discussed as barriers to engagement in DCS [20,48,77,108]:

*“They’ve got better things to do with their time than line up on some machine. They just want to get fixed, you know? And most of them don’t really care. Most of them are in some kind of state where, quite frankly, if they died they wouldn’t really care anyway. Or the thought of it doesn’t really scare them anymore, you know what I mean?”.*

[20] (p. 8)

Further, service user opinion of the drug market may also influence engagement. For example, trust in a particular seller can reduce the perceived necessity of DCS as people view the sellers as a credible source of information about the content of a drug [109]. Finally, it is likely that, regardless of an individuals’ overdose experience and harm reduction training, previous negative experiences with police and wider services can reduce likelihood of engagement due to a perception that the risks of engagement in DCS outweigh the benefits [46,51,83]. Such factors highlight that PWUD often exist in a complex risk environment with factors which mitigate the potential for previous negative experiences of drug use to translate into engagement with DCS.

## 4. Discussion and Recommendations

The synthesis of empirical findings using realist methodology has allowed a theoretical understanding of the drug checking process which, in turn, has provided insight into how the desired outcome of increased engagement might be achieved in future service development and implementation. Through in-depth exploration of the data, seven refined programme theories have been proposed, and the contexts, mechanisms, and outcomes within each theory discussed at length. By developing programme theories that provide comprehensive understanding of the intervention process on multiple levels, from the individual to wider policy and systems level, a deeper exploration of how socio-ecological factors interact to impact DCS implementation has been achieved. Our findings highlight how DCS should not be conceptualised as an individual level intervention, particularly given the contradictory data across the literature relative to individual-level contexts. Instead, future design and implementation must consider DCS to be an intervention which is significantly influenced by meso- and macro- level factors.

The review has shown that, at a macro level, whilst there appears to be growing support for the implementation of DCS across a range of stakeholders, government support and subsequent funding for such services remains highly variable [4,6]. Services which secure support and funding from central government are typically able to operate more securely and afford the necessary equipment and staffing costs, all of which may increase engagement in drug checking through enabling the provision of services which better meet service user needs. Given the increasing levels of drug-related harm and death globally [23], deploying interventions such as DCS as part of an urgent, public health response is arguably time critical. To progress with future DCS implementation, there is a clear need for further exploration of issues such as how different legislation, policy, and institutional contexts impact on frontline policing practices in relation to DCS, how this varies across contexts, and whether such practices are disproportionately targeting those from marginalised groups [39,75,128]. There is also a need for ongoing consideration of what we have termed enabling legislation to provide firmer assurances of protection for people accessing DCS. Although clear legislative guidance may provide a more coherent approach to DCS implementation, it is likely that services in many jurisdictions will continue to rely on less formal arrangements. It is important that further research is undertaken to identify how such arrangements are formed and maintained, and how they work in practice, particularly regarding any tensions between public health-oriented approaches to policing and expectations around law enforcement [59,129].

At an organisational level in regard to the testing process itself, equipment and methods impact the ability of DCS to meet service users’ needs and expectations. As noted, point-of-care testing methods often have substantial limitations in reliability and comprehensiveness of results, and the analytical sophistication of methods utilised by services varies widely [4,8,95]. Services that lack the infrastructure and funding for method development may be less able to meet the needs of service users and have to utilise less reliable and comprehensive testing methods. To facilitate improvements in equipment and methods systemically, governments should consider funding schemes which encourage the development of low-cost, easy to use methods which provide rapid, accurate results. These are traits which make drug checking equipment more suited for use in harm reduction settings and enable the scaling up of drug checking provision [121,130]. Additionally, although there is evidence that the provision of quantitative information is important for increasing engagement, there is also some indication that service users will still engage with more basic forms of testing, albeit potentially in lower numbers [10,86]. This is important given it is likely that many services will continue to operate in contexts without substantial funding from governments, utilising more limited testing methods. However, accurate quantitative data may provide service users with a chance to more accurately dose which could, in turn, reduce risk of overdose. Therefore, services should ideally aim to draw on available means to develop their testing methods, such as collaboration with existing DCS, universities, and supportive public health bodies, in order to provide the most comprehensive possible information to service users. Services can also look to additional sources of funding such as private bodies and donations with an interest in furthering harm reduction [15].

As well as the testing process, integration of DCS into existing services impacts levels of engagement in drug checking, an impact which will vary according to service user experiences [17,20], risk environment, patterns of use and several other factors. Put simply, different individuals will view different sites for integration as more or less accessible and appropriate. Much of the evidence on the impact of different integration sites comes from North America, where drug checking is typically delivered in low threshold, harm reduction sites with high levels of pre-existing footfall to access other interventions [58]. Such settings are argued to be the most appropriate sites for those with dependency on drugs, particularly those who could be considered at highest risk of experiencing drug-related harm. Alternatively, implementing community-based DCS in locations such as churches, community, and youth centres could be successful due to the ‘neutrality’ of those buildings compared to specific drug services and may appeal to a broad demographic [54]. To further aid with engagement, approaches such as outreach testing or sample collection, postal drug checking, or discrete sample drop-off points could be incorporated into services, depending on legislative arrangements [18,107,130]. Such methods may provide options for engagement amongst those for whom attendance at a fixed site is unfavourable, as well as offering relatively cost-effective means of expanding access to DCS [130].

As well as accessibility of the service itself, from a health-equity perspective, it is important for people with lived/living experience to be centrally involved in the design, delivery and evaluation of DCS. Our findings highlight that it is important that such involvement is not tokenistic: PWUD should be involved in key decision making and lead roles. This may, at times, be in tension with systems which are focused on managing risk through robust governance procedures, and it may be particularly challenging in countries such as the UK due to overarching and sometimes opposing demands from insurers and Home Office licensing regarding aspects such as qualifications, experience, and police checks which provide information about previous criminal convictions prior to employment. Regardless, it is important for drug checking to be perceived as a community service, rooted in the needs and experiences of PWUD, so if central involvement at all levels is not feasible in the initial introduction of DCS, this should be an explicit aim of services in future stages of development.

### Strengths and Limitations

A key strength of this study is its adherence to a theoretically informed, rigorous review framework. A realist approach is particularly appropriate for this inquiry where evidence is still emerging, and significant questions remain. A related limitation may be that some of the literature cited is based on research that is pre-or-early intervention and accordingly lacking evidence and robust outcome measures. Therefore, this study provides a foundational review of the emergence and growth of community DCS to inform future research by providing areas of focus and questions in relation to engagement outcomes. Consistent with the realist approach, there is a need for the programme theories outlined here to be tested and refined in future work, to further capture and reflect the contextual complexity in which DCS are delivered, and the subsequent impact of such factors on engagement. In particular, incorporation of quantitative data into the programme theories is recommended. Further, while grey literature was included as a priority in the review, we recognise that reviews are more likely to locate publications that include academics and researchers and may miss the evidence and knowledge of more grassroots and voluntary sector publications which are arguably the evidence-base for current community-based DCS. It is also important to note that the majority of included studies were from high-income countries which could limit transferability of findings to DCS in the Global South. There may be a growing number of DCS in these areas, but the lack of harm reduction infrastructure, and risk of criminalisation, imprisonment, and/or harsher punishment relating to drug use in the Global South may hinder implementation and/or reporting of harm reduction interventions [5]. The limitations inherent to realist methodology are important to note. While realist methodology attempts to show causation between intervention components, it is important to acknowledge that these predictions may be fallible [26]. This limitation is inherent to all realist research; therefore, the refined programme theories should be tested in future work in order to provide further evidence and/or refinement. Finally, realist reviews are based on guiding principles rather than standardised rules, so this can raise questions around subjective interpretations of the review process [25]. To address this, we have prioritised transparency throughout the review by submitting our protocol to PROSPERO, adhering to robust quality standards [33], involving several members of the research team in each stage of the review, and through thorough documentation and in-depth discussion of all key decisions.

## 5. Conclusions

In the context of increasing drug-related deaths in many countries, and increasingly unpredictable and potent unregulated drugs, community-based drug checking has been emerging as an important harm reduction tool. This review explored how community-based DCS could be designed and implemented to promote engagement amongst PWUD. Exploration of implementation effectiveness relative to community-based DCS has arguably been held back by a lack of funding, limited academic interest in the topic until recently, and pressure to focus on the effectiveness of DCS in affecting behavioural change, in order to secure the interventions acceptance amongst policy makers, and thus secure further funding [1]. This review has shown that there is a clear need for exploration into *how* community-based DCS operate within wider contexts, and how mechanisms discussed within each programme theory can be fostered. Approaching drug checking research with this lens could enrich understanding of the differences between DCS across jurisdictions, and the impact these differences may have on implementation, service delivery, and subsequent levels of engagement amongst PWUD.

## Figures and Tables

**Table 1:** Overview of the themes that constitute the IPTs as hypothesised by the research team.

IPT Number	IPT Theme
1	Policing—service user point of view
2	Policing—police point of view
3	Affected family and service user involvement in implementation
4	Lived experience central to the service
5	Service users’ previous experience with substances
6	Existing drug market
7	Location of service
8	Integration into existing services
9	Community stigma
10	Existing relationships with service staff
11	Available equipment and expectations of tests
12	Reach of service
13	Existing level of drug-related harm
14	Focus of service (i.e., pill testing vs. other types of testing)
15	Individual differences of service users

**Table 2:** Seven refined programme theories.

Programme Theory Number and Name (Including Sub-Theories)
PT1: Legislation and regulation *Sub-theory 1: Exemptions and service user risk* *Sub-theory 2: Exemptions and staff risk* *Sub-theory 3: Government and policing policy* *Sub-theory 4: On the ground policing practice*
PT2: Existing drug market and level of drug-related harm
PT3: Integration into an existing service *Sub-theory 1: Service staff point of view* *Sub-theory 2: Service user point of view*
PT4: Lived/living experience at all levels
PT5: Accessibility
PT6: Testing process *Sub-theory 1: Equipment and expertise availability* *Sub-theory 2: Service user expectations of DCS tests*
PT7: Service users’ previous experience

## Data Availability

This study was a review of existing data, which is openly available at locations cited in the reference section. No new data were created or analysed in this study.

## Supplementary Materials

The following supporting information can be downloaded at: https://www.mdpi.com/article/10.3390/ijerph191911960/s1. Supplementary File S1: IPT table; Supplementary File S2: Included studies table; Supplementary File S3: Acronym list.
