# Supplementary material for: A Realist Review of How Community-Based Drug Checking Services Could Be Designed and Implemented to Promote Engagement of People Who Use Drugs
Source: Int J Environ Res Public Health. 2022 Sep 22;19(19):11960. doi: 10.3390/ijerph191911960 (PMC9564958; doi:10.3390/ijerph191911960)
Supplement: Supplementary file 1 [file ijerph-19-11960-s001.zip › Supp File S1 - IPT table.pdf]

Supplementary File S1: Overview of the CMOcs that constitute the IPTs as hypothesised by the research team.

IPT number	IPT theme	Context	Mechanism	Outcome
1	Policing – service user point of view	Legal framework	Fear of criminalisation	Decreased engagement
2	Policing – police point of view	Transparent positionality from people in power such as government and police	Clarity and understanding from police of role and how DCS fit into legislation	Better partnership working and implementation and ability to communicate effectively to public
3	Affected family and service user involvement in implementation	Inclusive approach including families and PWUD at the centre of DCS	Feeling included and like the service is tailored for them, with their input, feeling their voices are heard	Increased engagement and buy-in
4	Lived experience central to the service	Lived experience central to the intervention	Services viewed as more trustworthy and/or reliable	Higher acceptance, engagement, and buy-in from potential service users, particularly marginalised groups
5	Service user's previous experience with substances	Previous experience (either self or witnessed) in overdose, or negative experience with contamination/adulteration, or has training in overdose, or has experience/knowledge of DCS	Increased awareness and knowledge of risk	Increased willingness to engage
6	Existing drug market	Existing warnings or prevalence of unexpected substances within drugs on the market	Understanding of increased need for testing to reduce harm	Increased engagement (service users) and accepted implementation plan (stakeholders)
7	Location of service	Outreach available<br><br>Increased number of points in different locations where a sample can be submitted	Services viewed as more trustworthy and/or reliable	Increased reach of service and increased engagement of diverse groups

8	Integration into existing services	Integration with/into existing services, although drug checking specific training also must be available for existing staff for this to work	For staff: increased understanding about how DCS can meet service user need<br><br>For service users: Less fearful of engaging due to decreased perception of stigma compared to stand alone services	Higher levels of engagement
9	Community stigma	Existing stigmatising views of substance use<br><br>Lack of privacy around services<br><br>Non-discreet services	Fear and/or experience of stigmatisation	Decreased engagement
10	Existing relationships with service staff	Client has existing link to harm reduction services and relationships with the staff there	Clients feel more comfortable discussing substance use<br><br>Increased trust	Increased engagement, particularly among marginalised groups<br><br>Increased acceptability of advice/results given
11	Available equipment and expectations of tests	Existing staff expertise<br><br>Existing service user desire to wait for/know info (in particular, this usually consists of a desire to know quantitative information).<br><br>Available equipment and ability to provide some level of quantitative outcome quickly<br><br>Ability of the test is clear, i.e. what it can test for and what the limitations are	Satisfaction with service<br><br>Belief that expectations have been met	Engagement and re-engagement more likely

12	Reach of service	Existence of effective partnership networks within cities	Increased working together, sharing information, communication, and existing set of procedures when sharing information/alerts	Greater reach of DCS and increased engagement across diverse groups
13	Level of drug-related harm	Level of drug-related harm in a country/city and specific issues within a city e.g. increased blood borne viruses (BBV), or patterns of drug use relating to certain drugs and/or certain groups	Increased understanding and motivation to work towards and push for evidence-based, holistic, harm reduction, wrap around interventions	Change in legislation, commitment, funding, or other structural change to create enabling environment for implementation and increased engagement
14	Focus of service	'Type' of testing i.e. testing associated with recreational use or testing geared towards drugs such as opioids, benzodiazepines, etc.	Clients feeling like the service is 'for them' and adequately meets their needs	Increased or decreased engagement
15	Individual differences of service users	Ethnicity, age, housing status, individual attitudes, previous experience, gender, sexuality	Fear of criminalisation, fears of violence; less desire to use service	Increased or decreased engagement
