# Supplementary material for: A Realist Review of How Community-Based Drug Checking Services Could Be Designed and Implemented to Promote Engagement of People Who Use Drugs
Source: Int J Environ Res Public Health. 2022 Sep 22;19(19):11960. doi: 10.3390/ijerph191911960 (PMC9564958; doi:10.3390/ijerph191911960)
Supplement: Supplementary file 1 [file ijerph-19-11960-s001.zip › Supp File S2 - included studies table.pdf]

#	Authors	Year	Title	Type of literature	Literature topic/subject	Jurisdiction	PT supported
1.	Aasen	2021	Drug checking: Shedding light on substance use issues	Blog article	*Drug checking:* Community-DCS	Canada	PT1, PT3, PT4, PT5, PT6
2.	Babor	2017	Development of services for substance use problems: need for a system-based approach	Literature review/conceptual piece	*Harm reduction/substance use*	Europe, Various jurisdictions	PT3
3.	Bacon	2021	Desistance from criminalisation: police culture and new directions in drugs policing	Policy review and based on previous qual work	*Legislation, policing and policy (drugs)*	UK	PT1
4.	Bardwell et al	2019	Implementation contexts and the impact of policing on access to supervised consumption services in Toronto Canada	Qualitative study: Interviews, Ethnographic methods	*SIS/DCRs/OPRs:* Policing	Canada	PT1, PT5
5.	Bardwell et al	2019	'Trusting the source: The potential role of drug dealers in reducing drug-related harms via drug checking'	Qualitative study, interviews	*Drug checking:* Drug sellers	Canada	PT3, PT7
6.	Bardwell et al	2019	We don't got that kind of time, man. We're trying to get high!": Exploring potential use of drug checking technologies among structurally vulnerable people who use drugs	Qualitative study, interviews	*Drug checking:* Feasibility/acceptability/willingness Community-DCS	Canada	PT1, PT3, PT5, PT6, PT7
7.	Barratt and Nadine	2016	Drug checking can track the nature and size of the discrepancy between self-report and actual drugs consumed	Commentary/ response	*Drug checking*	Australia	PT2, PT6
8.	Barratt and Ritter	2017	Commentary on Vrolijk: The paradox of the	Commentary	*Drug checking:* Risk communication, Market monitoring	Australia	PT1, PT2

			quality control problem				
9.	Barratt et al	2020	On-site drug checking can provide quantitative analysis	Commentary piece: response to Scott and Scott's article - 'pill testing at music festivals, is it evidence based harm reduction'	*Drug checking:* Equipment	Australia	PT6
10.	Barratt et al	2018	Global review of services operating in 2017	Review of DCS, surveys	*Drug checking:* Models of service delivery, Funding/policy, Equipment	Australia, Information from various jurisdictions (global)	PT2, PT5, PT6
11.	Barratt et al	2018	Pill testing or drug checking in Australia: Acceptability of service design features	Quantitative survey	*Drug checking:* Acceptability/feasibility/willingness<br>Service design	Australia	PT1, PT2, PT6
12.	Barratt et al	2018	Profiles of drug checking services in 2017	Review of DCS, surveys	*Drug checking:* Models of service delivery, Funding/policy, Equipment	Australia, Information from various jurisdictions (global)	PT6
13.	Beaulieu et al	2021	Is expected substance type associated with timing of drug checking service utilization?: A cross sectional study	Statistical analysis	*Drug checking:* Community-based DCS, Engagement	Canada	PT6
14.	Beaulieu et al	2020	Effect of witnessing an overdose on the use of drug checking services among people who use illicit drugs in Vancouver, Canada	Statistical analysis of cohort data	*Drug checking:* Engagement, Community-based DCS	Canada	PT2, PT5, PT7
15.	Beckett	2016	The uses and abuses of police discretion: toward harm reduction policing	Theoretical/historical overview	*Legislation, policing and policy (drugs)*	United States	PT1
16.	Benschop et al	2002	Pill Testing, Ecstasy and Prevention	Profiles of 3 drug checking services	*Drug checking:* Models of service delivery, Behavioural outcomes, Engagement	Europe, The Netherlands, Austria and Germany	PT3, PT7
17.	Betsos et al	2021	‘I couldn't live with killing one of my friends or anybody”: A	Qualitative research study, interviews	*Drug checking:* Drug sellers	Canada	PT1, PT2

			rapid ethnographic study of drug sellers' use of drug checking'				
18.	Betzler et al	2020	Drug checking and its potential impact on substance use	Questionnaire, analysis	*Drug checking:* Engagement	Europe, Germany	PT1
19.	Biggar et al	2021	Towards cross-Canada monitoring of the unregulated street drug supply	Commentary	*Drug market monitoring*	Canada	PT2, PT5
20.	Brunt	2017	Drug checking as a harm reduction tool for recreational users: opportunities and challenges	Policy report	*Drug checking:* Market monitoring, Equipment	Europe, The Netherlands, Various jurisdictions	PT1, PT2, PT6
21.	Brunt and Niesink	2011	The Drug information and Monitoring system (DIMS): Implementation, results, and international comparison	Analysis of DCS	*Drug checking:* Equipment, Market monitoring, Policy, Community-based DCS	Europe, The Netherlands	PT2
22.	Burkhart and Kriener	2003	Pill testing as selective prevention	Commentary	*Drug checking*	Europe, Spain	PT1, PT2
23.	Burris et al	2004	Addressing the 'risk environment' for injection drug users: the mysterious case of the missing cop	Literature review and theory	*Legislation, policing and policy (drugs)*	United States	PT1
24.	Buxton et al	2019	The British Columbia Drug Overdose and Alert Partnership: Interpreting and sharing timely illicit drug information to reduce harms	Analysis/review	*Drug market monitoring*	Canada	PT2, PT4
25.	Carroll	2021	Auras of detection: power and knowledge in drug prohibition	Theory/commentary, drawing on previous qualitative work	*Drug checking:* Community-DCS, Equipment, Policing	United States	PT1, PT2, PT4, PT6
26.	Carroll et al	2022	The Bronze Age of Drug Checking: Barriers and facilitators to	Qualitative study, interviews and case study	*Drug checking:* Equipment Service implementation, legal challenges, Policing,	United States	PT1, PT2, PT3, PT6

			implementing advanced drug checking amidst police violence and Covid-19		Community based DCS		
27.	CBC Radio	2021	How a new drug-checking kit could turn smartphones into overdose prevention tools	News article	*Drug checking:* Equipment, Community-based DCS	Canada	PT6
28.	Davis et al	2022	Legality of drug checking equipment in the US: A systematic legal analysis	Systematic literature review	*Drug checking*: Equipment/Paraphernalia, Legal challenges.	United States	PT1
29.	Deconinck et al	2020	An infrared spectroscopic approach to characterise white powders, easily applicable in the context of drug checking, drug prevention and on-site analysis	Validation of equipment/methods	*Drug checking:* Equipment	Europe, Belgium	PT6
30.	Dolan and Johnston	2020	The Drug purity discounting task	Experimental research	*Drug checking:* Behavioural outcomes	United States	PT2, PT7
31.	Fregonese et al	2021	Drug checking as a strategy for harm reduction in recreational contexts: Evaluation of two different drug analysis methodologies	Validation of equipment/methods	*Drug checking:* Equipment	Europe, Italy	PT1, PT2, PT6
32.	Gine	2019	Drug checking services: An essential tool for risk and harm reduction	Commentary	*Drug checking*	Europe, Spain	PT1, PT2, PT6
33.	Glick et al	2019	Stakeholder perspectives on implementing fentanyl drug checking: Results from a multi-site study	Qualitative study: interviews	*Drug checking:* Acceptability/feasibility/willingness Community-based DCS	United States	PT5, PT3, PT6
34.	Goncalves et al	202	Suitability of infrared spectroscopy for drug checking in harm reduction centres	Validation of equipment methods	*Drug checking:* Community-based DCS, Equipment	Europe, France	PT2, PT3, PT6
35.	Goldman et al	2019	Perspectives on rapid fentanyl test strips as a harm reduction	Mixed methods, Quantitative survey and	*Drug checking:* Equipment, Acceptability/feasibility/willingness	United States	PT1, PT6,

			practice among young adults who use drugs: A qualitative study	qualitative interviews	Engagement, Community-based DCS		
36.	Gozdzialski et al	2021	Fentanyl detection and quantification using portable Raman spectroscopy in community drug checking'	Validation of equipment/methods	*Drug checking: Equipment, Community-based DCS*	Canada	PT6
37.	Green et al	2020	An assessment of the limits of detection, sensitivity and specificity of three devices for public-health based drug checking of fentanyl in street acquired samples	Validation of equipment/methods	*Drug checking:* Equipment	United States	PT1, PT3, PT4, PT6
38.	Greer et al	2022	Simple possession as a 'tool': Drug law enforcement practices among police officers in the context of depenalization in British Columbia, Canada	Qualitative study: interviews	*Legislation, policing and policy (drugs)*	Canada	PT1
39.	Grossman	2021	Five years into the overdose crisis, Vicoria's street drugs are stronger than ever	News article	*Drug checking:* Community-based DCS	Canada	PT2, PT6
40.	Groves	2018	Worth the test?' Pragmatism, pill testing and drug policy in Australia	Policy analysis	*Drug checking:* Festival DCS Policy/legislation	Australia	PT1
41.	Guirguis et al	2020	Piloting the UK's First Home-Office-Licensed Pharmacist-Led Drug Checking Service at a Community Substance Misuse Service	Service evaluation	*Drug checking:* Community-based DCS, Engagement, HO licensing, Equipment	UK	PT1, PT2, PT3, PT6
42.	Guirguis et al	2020	What about drug checking? Systematic review and netnographic	Analysis of social media content	*Drug checking:* Public/political debate	UK	PT1, PT2, PT3, PT5

			analysis of social media				
43.	Harper et al	2017	An overview of forensic drug testing methods and their suitability for harm reduction point of care services	Summary of equipment	*Drug checking:* Community-based DCS, Equipment	Canada	PT2, PT6
44.	Herbert et al	2018	Policing social marginality: contrasting approaches	Theoretical	*Legislation, policing and policy (drugs)*	United States	PT1
45.	Hungerbuehler et al	2011	Drug Checking: A prevention measure for a heterogeneous group with high consumption frequency and polydrug use - evaluation of zurich's drug checking services	Service evaluation/questionnaire	*Drug checking:* Festival DCS, Engagement.	Europe, Switzerland	PT3, PT7
46.	Karamouzian et al	2018	Evaluation of a fentanyl drug checking service for clients of a supervised injection facility, Vancouver, Canada	Service evaluation	*Drug checking:* Community-based DCS, Equipment, Engagement, Behavioural outcomes	Canada	PT1, PT2, PT3, PT6
47.	Karch et al	2021	Results from a mobile drug checking pilot program using three technologies in Chicago, IL, USA	Validation of equipment/method	*Drug checking:* Equipment Community-based DCS Mobile DCS	United States	PT1, PT2, PT5, PT6
48.	Kennedy et al	2019	Peer worker involvement in low-threshold supervised consumption facilities in the context of an overdose epidemic in Vancouver, Canada	Qualitative study: Qualitative interview, Ethnography	*SIS/DCRs/OPRs:* Peer workers	Canada	PT2, PT5, PT4,
49.	Kennedy et al	2018	Willingness to use drug checking within future supervised injection services among people who inject drugs in a	Quantitative survey	*Drug checking:* Community based DCS, Acceptability/feasibility/willingness	United States	PT2, PT5, PT6

			mid-sized Canadian city				
50.	Kerr and Tupper	2017	Drug checking as a harm reduction intervention: Evidence review report	Evidence review/report	*Drug checking:* Equipment, Community-based DCS	Canada	PT1, PT2, PT6
51.	King	2015	Facilitate recreational drug test to help save lives	Blog/opinion piece	*Drug checking:* Festival-DCS	UK	PT2
52.	KnowYourStuffNZ	2021	Drug and substance checking legislation bill passes into law	News/blog article	*Drug checking:* Legislation	New Zealand	PT1
53.	Komesaroff and Lloyd Jones	2019	Pill Testing warrants assessment in careful pilot programs	Editorial/commentary	*Drug checking:* Festival-DCS	Australia	PT2
54.	Kosmicare	2020	Drug checking report Nov 2019-Nov 2020	DCS Annual Report	*Drug checking:* Engagement, Community-based DCS.	Europe, Portugal	PT2
55.	Krieger et al	2018	High willingness to use rapid fentanyl test strips among young adults who use drugs	Quantitative survey	*Drug checking:* Equipment, Acceptability/feasibility/willingness	United States	PT3, PT6, PT7
56.	Krieger et al	2018	Use of rapid fentanyl test strips among young adults who use drugs	Quantitative survey	*Drug checking:* Equipment, Engagement	United States	PT3, PT6, PT7
57.	Kriener et al	2001	An inventory of on-site pill testing interventions in the EU	Inventory/survey	*Drug checking:* Models of service delivery	Europe, Various jusrisdictions	PT1, PT2, PT4, PT5
58.	Koning et al	2021	Visitors of the Dutch drug checking services: Profile and drug use experience	Quantitative survey	*Drug checking:* Community-based DCS, Client demographics, Engagement	Europe, The Netherlands	PT3, PT7
59.	Laing et al	2021	An outbreak of novel psychoactive substance benzodiazepine in the unregulated drug supply: preliminary results from a community drug checking program using point of care and	Validation of equipment/methods	*Drug checking:* Community-based DCS, Equipment	Canada	PT2, PT6

			confirmatory methods				
60.	Laing et al	2018	Drug checking as a potential strategic overdose response in the fentanyl era	Commentary	*Drug checking:* Community-based DCS	Canada	PT1, PT6
61.	Larnder et al	2021	Third party drug checking: accessing harm reduction services on behalf of others	Statistical analysis	*Drug checking:* Community-based DCS, Engagement	Canada	PT1, PT7
62.	Lianping et al	2020	Detecting fentanyl using point of care drug checking technologies: a validation study	Validation of equipment/methods	*Drug checking:* Community-based DCS Equipment	Canada	PT6
63.	Lianping et al	2021	Detection of synthetic cannabinoid adulteration in the unregulated drug supply of three Canadian settings	Validation of equipment/methods	*Drug checking:* Community-based DCS Equipment	Canada	PT6
64.	Long et al	2020	Factors associated with drug checking service utilisation among people who use drugs in a Canadian setting	Statistical analysis	*Drug checking:* Community-based DCS, Engagement	Canada	PT3, PT5, PT6, PT7
65.	Maghsoudi	2018	Comparing models of drug checking services in Canada	Short document detailing equipment used in DCS	*Drug checking:* Equipment, Models of service delivery	Canada	PT2, PT6
66.	Maghsoudi et al	2021	Drug checking services for people who use drugs: a systematic review	Systematic review	*Drug checking:* Market monitoring Behavioural outcomes	Canada, Sources from multiple jurisdictions	PT2, PT3, PT5
67.	Maghsoudi et al	2020	Evaluating networked drug checking services in Toronto, Ontario: study protocol and rationale	Evaluation protocol	*Drug checking:* Community-dcs	Canada	PT2, PT3
68.	McCann and Vadivelu	2019	Saving Lives. Changing Lives. Summary Report on the findings from an	Service evaluation report	*OPR/Drug checking:* Engagement, Community-based DCS, Model of service delivery.	Canada	PT1, PT3, PT5,

			Evaluation of London's Temporary Overdose Prevention Site (TOPS)				
69.	McCrae et al	2020	Assessing the limit of detection of Fourier-transform infrared spectroscopy and immunoassay strips for fentanyl in a real world setting	Validation of equipment/methods	*Drug checking:* Community-based DCS Equipment	Canada	PT2, PT6
70.	McCrae et al	2020	The effect of injecting alone on the use of drug checking services among people who inject drugs	Statistical analysis	*Drug checking:* Engagement Community-based DCS	Canada	PT1, PT5, PT7
71.	McCrae et al	2019	Drug checking: Operational technician manual	Manual for practitioners	*Drug checking:* Equipment.	Canada	PT1, PT2, PT6
72.	Measham	2020	City checking: Piloting the UK's first community-based drug testing (drug checking) service in 2 city centres	Evaluation/analysis of pilot data	*Drug checking:* Engagement, Models of service delivery, Behavioural outcomes, Community-based DCS	UK	PT1, PT2, PT3, PT6, PT7
73.	Measham	2019	Drug safety testing, disposals and dealing in an English field: Exploring the operational and behavioural outcomes of the UK's first onsite 'drug checking' service	Evaluation/analysis of pilot data	*Drug checking:* Engagement, Behavioural outcomes. Festival DCS	UK	PT1, PT2, PT6
74.	Measham and Turnbull	2021	Intentions, actions and outcomes: A follow up survey on harm reduction practices after using an English festival drug checking service	Quantitative survey/DCS data	*Drug checking:* Engagement, Behavioural outcomes Festival DCS	UK	PT2, PT6, PT7

75.	Miller et al	2016	Drug testing, accuracy and harm reduction	Response to Barratt and Ezard	*Drug checking:*<br>Festival DCS,<br>Equipment	Australia	PT2, PT6
76.	Moore	2004	Governing street-based injecting drug users: a critique of heroin overdose prevention in Australia	Ethnographic study	*Harm reduction and behaviour, risk environment*	Australia	PT1, PT3, PT5, PT7
77.	Morgan and Jones	2019	Pill testing as a harm reduction strategy: time to have the conversation	Commentary piece	*Drug checking:*<br>Festival DCS	Australia	PT2
78.	Mistler et al	2021	Engagement in Harm Reduction Strategies After Suspected Fentanyl Contamination Among Opioid-Dependent Individuals	Quantitative cross-sectional survey	*Harm reduction behaviours, PWUD*	United States	PT3, PT5, PT6, PT7
79.	Oh et al	2020	Fentanyl self-testing in a community-based sample of people who inject drugs, San Francisco	Quantitative survey	*Drug checking:*<br>Community-based DCS<br>Equipment,	United States	PT2, PT3, PT7
80.	Olding et al	2020	A low barrier and comprehensive community-based harm reduction site in Vancouver, Canada	Briefing from service evaluation	*Drug checking/harm reduction:*<br>Model of service delivery,<br>Community-based DCS	Canada	PT2, PT3
81.	Palamar et al	2019	Adulterants and altruism: A qualitative investigation of "drug checkers" in North America	Qualitative study, interviews	*Drug checking:*<br>Legislation<br>Motivation for drug checking	United States	PT1, PT4, PT6, PT7
82.	Park et al	2021	Evaluation of fentanyl test strip distribution in two Mid-Atlantic syringe services programs	Quantitative survey	*Drug checking:*<br>Equipment<br>Acceptability/feasibility/willingness<br>Community-based DCS	United States	PT2, PT6
83.	Park et al	2020	A fentanyl test strip intervention to reduce overdose risk among female sex workers	Quantitative survey	*Drug checking:*<br>Equipment<br>Acceptability/feasibility/willingness<br>Community-based DCS	United States	PT2, PT6

			who use drugs in Baltimore: Results from a pilot study’				
84.	Peiper et al	2019	Fentanyl test strips as an opioid overdose prevention strategy: Findings from a syringe services program in the South-eastern United States	Quantitative survey	*Drug checking:*<br>Equipment<br>Behavioural outcomes<br>Community-based DCS	United States	PT2, PT6
85.	Ramsay et al	2021	Fentanyl quantification using portable infrared spectroscopy. A framework for community drug checking	Validation of equipment/results	*Drug checking:*<br>Community-based DCS<br>Equipment	Canada	PT6
86.	Reed et al	2021	“I probably got a minute”: Perceptions of fentanyl test strip use among people who use stimulants	Qualitative study, interview	*Drug checking:*<br>Community-based DCS<br>Equipment<br>Acceptability/feasibility/willingness	United States	PT1, PT6
87.	Ritter	2020	Making drug policy in summer - drug checking in Australia as providing more heat than light	Discourse/policy analysis	*Drug checking:*<br>Public/political debate	Australia	PT2
88.	Rouhani et al	2019	Harm reduction measures employed by people using opioids with suspected fentanyl exposure in Boston, Baltimore, and Providence	Quantitative cross-sectional survey	*Drug checking:*<br>Behavioural outcomes<br>Community-based DCS	United States	PT1, PT6, PT7
89.	Sage et al	2021	Drug checking manual: Creating safer spaces with harm reduction in drug checking settings	Manual/how to for DCS	*Drug checking:*<br>Practical considerations<br>Safe spaces<br>Lived experience	Canada	PT1, PT3, PT4
90.	Sande and Sabic	2018	The importance of drug checking outside the context of nightlife in Slovenia	Quantitative survey	*Drug checking:*<br>Engagement,<br>Community-based DCS.	Slovenia	PT2, PT3, PT6
91.	Schisler	2021	A new device that tests drugs	News article	*Drug checking:*<br>Community-based DCS,	Canada	PT6

			in minutes boosted by province amongst B.C.s overdose crisis		Equipment		
92.	Schneider et al	2016	Pill testing at music festivals: can we do more harm?	Editorial	*Drug checking:* Festival DCS.	Australia	PT2, PT6
93.	Scott and Henderson	2021	Commentary on Andersen et al.: Time for drug checking for heroin users?	Commentary	*Drug checking:* Community-based DCS	UK	PT2, PT3
94.	Scott and Scott	2020	Author reply to the neglected benefits of drug checking for harm reduction	Response to Smit-Rigter and Van Der Gouwe	*Drug checking:* Festival DCS	Australia	PT2, PT6
95.	Scott and Scott	2020	Author reply to Tremonti et al	Response to Tremonti et al's comment on the authors paper	*Drug checking:* Festival DCS	Australia	PT6
96.	Scott and Scott	2929	Comment on pill testing review reply	Response	*Drug checking:* Festival DCS	Australia	PT6
97.	Scott and Scott	2020	Pill testing at music festivals: is it evidence-based harm reduction?	Narrative review	*Drug checking:* Festival DCS	Australia	PT6, PT7
98.	Scottish Government	2021	International approaches to drug law reform	Policy report Literature review	*Legislation, policing and policy (drugs)*	Scotland, International evidence from multiple countries	PT1, PT2
99.	Shannon et al	2008	Mapping violence and policing as an environmental-structural barrier to health service and syringe availability among substance-using women in street-level sex work	Social mapping using questionnaires and statistical analysis	*Legislation, policing and policy (drugs)*	Australia	PT1, PT7
100	Sherman et al	2018	Fentanyl overdose reduction checking analysis study	Report/evaluation	*Drug checking:* Equipment, Engagement, Behavioural outcomes, Models of service delivery, Community-based DCS.	United States	PT3, PT6, PT7
101	Sherman et al	2019	Acceptability of implementing community-based drug checking	Quantitative questionnaire	*Drug checking:* Community-based DCS, Equipment, Acceptability/feasibility/willingness	United States	PT2, PT3, PT6

			services for people who use drugs in three United States cities: Baltimore, Boston and Providence				
102	Skrypnek	2021	Three years in: Vancouver Island Drug Checking Project working to save lives	News article	*Drug checking:* Community-based DCS	Canada	PT2
103	Smit-Rigter and Van Der Gouwe	2020	The neglected benefits of drug checking for harm reduction	Letter to editor	*Drug checking:* Community-based DCS	Europe, The Netherlands	PT6
104	Smit-Rigter and Van Der Gouwe	2019	The Drugs Information and Monitoring System (DIMS) factsheet	Factsheet	*Drug checking:* Community-based DCS, Models of service delivery	Europe, The Netherlands	PT1, PT4, PT6
105	Soukup-Baljak et al	2015	Drug quality assessment practices and communication of drug alerts among people who use drugs	Questionnaires and focus groups	*Risk communication, harm reduction, drug alerts*		PT2, PT7
106	SSA	2021	Introduction to the first Home Office-licensed ‘drug checking’ service: the SSA talks to Dr. Amira Guirguis	Interview (Amira Guirguis)	*Drug checking:* Community-based DCS, Equipment	UK	PT1, PT3, PT6
107	Sumnall and Atkinson	2022 (in press)	Drug safety communication for drug checking services	Evidence review/policy report	*Drug checking:* Risk communication	UK, Evidence from multiple jurisdictions	PT2, PT3
108	TEDI	2011	Factsheet on drug checking in Europe	Factsheet	*Drug checking:* Models of service delivery	Europe, Evidence from multiple jurisdcitions	PT1, PT2, PT3, PT4, PT6
109	Tissot	2017	Promoting harm reduction strategies by means of drug checking: its use in CAARUD	Book chapter, ethnography/theory	*Harm reduction*	Europe, France	PT4, PT6
110	Tobias et al	2021	Drug checking identifies counterfeit alprazolam tablets	Validation of equipment/methods	*Drug checking:* Community-based DCS, Equipment	Canada	PT6
111	Trayner et al	2021	High Willingness to use drug consumption	Quantitative survey	*SIS/DCR/OPR:* Acceptability/feasibility/willingness	Scotland, UK	PT1, PT2, PT3

			rooms among people who inject drugs in Scotland: findings from a national bio-behavioural survey among people who inject drugs				
112	Tregoning	2016	Drug checking services - Brief for UnHarm	Drug checking brief	*Drug checking*	Australia	PT1, PT5
113	Tremonti et al	2020	Comment on pill testing review	Reply to article by Scott and Scott (2020)	*Drug checking:*<br>Festival based-DCS	Australia	PT2
114	Tupper et al	2018	Initial results of a drug checking pilot program to detect fentanyl adulteration in a Canadian setting	Validation of equipment	*Drug checking:*<br>Equipment,<br>Engagement,<br>Community-based DCS	Canada	PT1, PT6
115	Valente et al	2019	Evaluation of a drug checking service at a large scale electronic music festival in Portugal	Quantitative survey, evaluation	*Drug checking:*<br>Festival DCS,<br>Behavioural outcomes	Europe, Portugal	PT3, PT6
116	Ventura et al	2012	Drug Checking Service Good Practice Standards	Good practice standards DCS	*Drug checking:*<br>Models of service delivery	Europe, Brussels, Multiple jurisdcitions	PT1
117	Vrolijk et al	2017	Is online information on ecstasy tablet content safe?	Analysis of online information	*Drug market monitoring*	Europe, The Netherlands	PT2
118	Wallace et al	2021	Equity oriented frameworks to inform responses to opioid overdoses: a scoping review	Literature scoping review	*Overdose response/harm reduction:*<br>Health equity	Canada	PT4
119	Wallace et al	2021	Implementing an integrated multi-technology platform for drug checking: Social, scientific, and technological considerations	Qualitative study: interviews	*Drug checking:*<br>Communicating risk,<br>Models of service delivery,<br>Equipment,<br>Community-based DCS.	Canada	PT3, PT5, PT6
120	Wallace et al	2021	The potential impacts of community drug checking within the overdose crisis:	Qualitative study: interviews	*Drug checking:*<br>Community-based DCS,<br>Behavioural outcomes,<br>Market monitoring,<br>policy	Canada	PT2

			qualitative study exploring the perspective of prospective service users				
121	Wallace et al	2020	What is needed for implementing drug checking services in the context of the overdose crisis? A qualitative study to explore perspectives of potential service users	Qualitative study: interviews	*Drug checking:* Acceptability/feasibility/willingness Community-based DCS, Models of service delivery	Canada	PT1, PT3, PT4, PT5, PT6
122	Watson et al	2018	Creating and sustaining co-operative relationships between supervised injection sites and police: A qualitative interview study of international stakeholders	Qualitative study, interviews	*SIS/DCRs/OPRs:* Policing	Canada, Various	PT1
123	Weatherston et al	2020	An unquantified uncertainty visualisation design space during the opioid crisis	Design	*Drug checking:* Community-based DCS, Communicating risk/uncertainty	Canada	PT6
124	Weicker et al	2020	Agency in the fentanyl era: Exploring the utility of fentanyl test strips in an opaque drug market'	Qualitative study, interviews	*Drug checking:* Equipment	United States	PT1, PT2, PT6, PT7
125	Winstock et al	2001	Ecstasy pill testing: harm minimization gone too far?	Report/commentary	*Drug checking:* Festival DCS	UK	PT1, PT2
126	Wood et al	2015	Aligning policing and public health promotion: insights from the world of foot patrol	Mixed methods: Randomized control trial and qualitative methods (interviewing, participant observation)	*Legislation, policing and policy (drugs)*	United States	PT1
127	Yates	2019	Drugs checker	Blog article	*Drug checking:* Festival-DCS	UK	PT1, PT2, PT6
128	Zibbell et al	2021	Consumer discernment of fentanyl in illicit	Quantitative survey	*Drug checking:* Community-based DCS, Equipment	United States	PT2, PT3, PT6,

			opioids confirmed by fentanyl test strips: Lessons from a syringe services program in North Carolina				
129	Maghsoudi and McDonald (webinar presenters)	2021	Toronto Drug checking service (Research's roundtable)	Webinar	*Drug checking:* Community-DCS, Policy, Equipment	Canada	PT1, PT2, PT3, PT4, PT6
130	Notes from the research team	2021	Meeting notes from conversations with DIMS staff	Research notes/informant notes	*Drug Checking:* Community-based DCS, Equipment, Policing	Europe, Netherlands	PT1, PT3, PT4, PT6
131	Notes from the research team	2021	Meeting notes from Scottish stakeholders	Research notes	*Drug checking:* Equipment, Models of service delivery, Policy/funding, Policing	Scotland, international evidence	PT1, PT2, PT3, PT4, PT5, PT6
132	Various authors	2019	APSAD 2019 conference, 10-13 November, Hobart, Australia	Conference abstracts	*Multiple topics in drug policy*	Australia	PT3
133	Various speakers	2021	Alliance of collaborative drug checking conference 2021	Conference notes	*Drug checking:* Community-based DCS, various subjects	Canada, United States, Mexico	PT1, PT2, PT3, PT4, PT5, PT6
