# Supplementary material for: A Realist Review of How Community-Based Drug Checking Services Could Be Designed and Implemented to Promote Engagement of People Who Use Drugs
Source: Int J Environ Res Public Health. 2022 Sep 22;19(19):11960. doi: 10.3390/ijerph191911960 (PMC9564958; doi:10.3390/ijerph191911960)
Supplement: Supplementary file 1 [file ijerph-19-11960-s001.zip › Supp File S3 - acronym list .pdf]

Supplementary File S3: Acronym list.

AIDs – acquired immunodeficiency syndrome

CMO – context mechanism outcome

CMOcs – Context mechanism outcome configuration

DBS – Disclosure and Barring Service

DCS – Drug checking service

DIMS – The Drugs Information and Monitoring System

DUID – driving under the influence of drugs

FTIR – Fourier-transform infrared spectroscopy

HIV – human immunodeficiency virus

IPED – image and performance enhancing drugs

IPTs – Initial programme theories

MDMA – 3,4-Methylenedioxymethamphetamine

PWUD – People who use drugs

UK – United Kingdom

US – United States
